# Long-Range Dependence in Financial Markets: A Moving Average Cluster Entropy Approach

**DOI:** 10.3390/e22060634

**Published:** 2020-06-08

**Authors:** Pietro Murialdo, Linda Ponta, Anna Carbone

**Affiliations:** 1 Institute of Condensed Matter Physics and Complex Systems, DISAT, Politecnico di Torino, 10129 Torino, Italy; 2 School of Industrial Engineering, LIUC-Università Cattaneo, Castellanza, VA 21052, Italy

**Keywords:** cluster-entropy, Shannon-entropy, financial markets, time series, dynamics

## Abstract

A perspective is taken on the intangible complexity of economic and social systems by investigating the dynamical processes producing, storing and transmitting information in financial time series. An extensive analysis based on the *moving average cluster entropy* approach has evidenced market and horizon dependence in highest-frequency data of real world financial assets. The behavior is scrutinized by applying the moving average cluster entropy approach to long-range correlated stochastic processes as the Autoregressive Fractionally Integrated Moving Average (ARFIMA) and Fractional Brownian motion (FBM). An extensive set of series is generated with a broad range of values of the Hurst exponent *H* and of the autoregressive, differencing and moving average parameters p,d,q. A systematic relation between moving average cluster entropy and long-range correlation parameters *H*, *d* is observed. This study shows that the characteristic behaviour exhibited by the horizon dependence of the cluster entropy is related to long-range positive correlation in financial markets. Specifically, long range positively correlated ARFIMA processes with differencing parameter d≃0.05, d≃0.15 and d≃0.25 are consistent with moving average cluster entropy results obtained in time series of DJIA, S&P500 and NASDAQ. The findings clearly point to a variability of price returns, consistently with a price dynamics involving multiple temporal scales and, thus, short- and long-run volatility components. An important aspect of the proposed approach is the ability to capture detailed horizon dependence over relatively short horizons (one to twelve months) and thus its relevance to define risk analysis indices.

## 1. Introduction

In recent years, much effort has been spent on studying complex interactions in financial markets by means of information theoretical measures from different standpoints. The information flow can be probed by observing a relevant quantity over a certain temporal range (e.g., price and volatility series of financial assets). Socio-economic complex systems exhibit remarkable features related to patterns emerging from the seemingly random structure in the observed time series, due to the interplay of long- and short-range correlated decay processes. The correlation degree is intrinsically linked to the information embedded in the patterns, whose extraction and quantification add clues to the underlying complex phenomena [1,2,3,4,5,6,7,8,9,10,11,12,13,14].

An information measure S(x) was proposed by Claude Shannon to the aim of quantifying the degree of uncertainty of strings of elementary random events in terms of their probabilities [15]. The elementary stochastic events are related to a relevant variable *x* whose values are determined by the probability $\left\{p_{i}\right\}$. For example, the size *ℓ* of a string (block), corresponding to a particular realization within the sequence, can be associated to the probability $p_{i}\left(\ell \right)$ that, for stationary processes, does not depend on the actual position of the string (block) in the sequence. The Shannon measure is then given by the expectation value $S\left(\ell \right)=\sum_{i}p_{i}\left(\ell \right)\log p_{i}\left(\ell \right)$ and is calculated over all possible strings *ℓ*. The *entropy density* is defined as $s_{\ell }=\lim_{\ell \to \infty }S(\ell )/\ell$ and quantifies the rate at which the process produces unexpected information as a function of the size *ℓ*.

A complexity measure K(x) to quantify the amount of information contained in the string *x* was proposed by Kolmogorov [16]. The relation between Kolmogorov complexity and Shannon entropy has been extensively investigated, in particular the entropy density $s_{\ell }$ for a stationary process corresponds to the Kolmogorov entropy rate [17].

The first step required for the practical implementation of entropy and complexity measures is a suitable partition of the sequence which is critical to unbundle random and deterministic blocks of given length (decryption). The method usually adopted for partitioning a sequence and estimating its entropy is based on a uniform division in blocks with same length [18,19,20,21].

The *cluster entropy method* [9,10,11] implements the partition via a moving average process. The *clusters* correspond to blocks of different sizes, defined as the portion between consecutive intersections of a given time series and moving average. The *cluster entropy method* has been applied to financial markets in [22,23]. Cumulative information measures (indexes) have been worked out with the ability to provide deep insights on heterogeneity and dynamics. In particular:

- **Heterogeneity**. Volatility series have been analysed by using the cluster entropy approach over a constant temporal horizon (six years of tick-by-tick data sampled every minute). An information measure of heterogeneity, the *Market Heterogeneity Index*I(T,n), where *T* and *n* are respectively the volatility and moving average windows, has been developed by integrating the cluster entropy curves of the volatility series over the cluster length τ. It has been also shown that the *Market Heterogeneity Index* can be used to yield the weights of an efficient portfolio as a complement to Markowitz and Sharpe traditional approaches for markets not consistent with Gaussian conditions [22].
- **Dynamics**. Prices series have been investigated by using the cluster entropy approach over several temporal horizons (ranging from one to twelve months of tick-by-tick data with sampling interval between 1 up to 20 seconds depending on the specific market). The study has revealed a systematic dependence of the cluster entropy over time horizons in the investigated markets. The *Market Dynamic Index*I(M,n), where *M* is the temporal horizon and *n* is the moving average window, defined as the integral of the cluster entropy over τ, demonstrates its ability to quantify the dynamics of assets’ prices over consecutive time periods in a single figure [23].

The present study is motivated by the results obtained in [23] showing that cluster entropy of real-world financial markets (NASDAQ, DJIA and S&P500) exhibits significant *market and horizon dependence*. According to classical financial theories, subsequent price deviations are identically and independently distributed (*iid*) and all the information are immediately reflected into markets, thus hampering past observations to predict future outcomes. If that were true, correlation would be negligible and prices would be simply modelled in terms of *fully uncorrelated Brownian motion*. However, several studies have shown that real world markets only partially behave according to the standard theory of perfectly informed and rational agents.

Here, we add further clues to the microscopic origin of the horizon dependence of the cluster entropy in financial markets. To this purpose, the cluster entropy approach is applied to an extensive set of artificially generated series with the aim of shedding light on the characteristic behaviour of real world assets [23]. We report results of the cluster entropy in *Geometric Brownian Motion* (GBM), *Generalized Autoregressive Conditional Heteroscedastic* (GARCH), *Fractional Brownian Motion* (FBM) and *Autoregressive Fractionally Integrated Moving Average* (ARFIMA) processes. Those are well-known processes characterized either by hyperbolically decaying or exponentially decaying correlation functions, features reflected in long-range or short-range dependent dynamics of the elementary random events. The performance of the *Autoregressive Fractionally Integrated Moving Average* (ARFIMA) process and its variants are receiving a lot of attention and are under intense investigation in the financial research community [24,25,26,27,28]. This work clearly demonstrates the relationship between the endogenous dynamics of the time series and their long-range dependence.

It is shown that deviations of the moving average cluster entropy behaviour in comparison to simple Brownian motion is unequivocally related to the long-range dependence of real-world market series. In particular, moving average cluster entropy results obtained on Fractional Brownian Motion with Hurst exponent *H* in the range 0≤H≤0.5 (negatively correlated series) show no time horizon dependence. Conversely, moving average cluster entropy results with Hurst exponent *H* in the range 0.5≤H≤1 (positively correlated series) exhibit some dispersion in the horizon dependence in analogy with the real-world financial markets. Results obtained on ARFIMA series confirm and extend the findings reported for FBMs. Horizon dependence of the cluster entropy is observed for a differencing parameter 0≤d≤0.5. Fine tuning of the horizon dependence is obtained by varying the autoregressive *p* and moving average *q* components in the ARFIMA series.

The low-frequency volatility has been identified as the long-run component to describe market dynamic fundamentals in recent works [29,30,31,32,33]. The current work demonstrates the ability of the cluster entropy to capture short-range and long-range variability in price returns, thus to identify short-run and long-run factors in volatility and their linkages with macroeconomic variables and asset prices. On account of the dispersion of the *Market Dynamic Index* I(M,n) at increasing values of the horizon *M*, our findings confirm that the slowest dynamic components (slowly evolving market fundamentals) reflect in the lowest-frequency volatility (large *M* scales) components of the assets. In this context, volatility can be modelled as a time dependent function, for example through the introduction of a quadratic spline to provide a smooth and nonlinear long-run trend in the volatility time series in the spline-GARCH model [29].

ARFIMA and Spline-GARCH belong to the class of *free-parameters model*, as they require for example the quadratic form of the time-dependence function parameters, or the autoregressive parameters. Conversely, the cluster entropy approach does not require free parameters. The cluster entropy is a *parameter-free model* based on data over some temporal horizons of choice. Hence, the comparison between results obtained by ARFIMA, GARCH models and those obtained by the cluster entropy approaches do not imply redundancy in the outcomes and is robust by design. The ability to extract market dynamic dispersion based only on data could be of relevance to disentangle performance of the different models at short and long horizons. This could be the case of ARFIMA models that tend to perform better on estimating asset variance at long-horizons compared to ARMA models that conversely produce superior results at short-horizons (see for example [24] where results of S&P500 are also reported).

In this work, the method is applied to mainstream financial assets as NASDAQ, DJIA and S&P500 tick-by tick data over the year 2018. The choice of these assets derives mainly from the need to validate the newly proposed cluster entropy approach on widely studied markets whose long range dependence has been quite widely investigated and broadly assessed by several studies. Further to these markets, interesting developments can be envisioned in different sectors that are strongly affected by macroeconomic variables and shock (e.g., time dependent variance and persistence have been observed in Real Estate securities [30] highlighting linkages between real estate stocks and market fundamentals, related to endogenous dynamics and horizon dependence).

The organisation of the work is as follows. The cluster entropy method used for the analysis and the investigated market and artificial data are described in Section 2. Results on cluster entropy and market dynamic index estimated over *Geometric Brownian Motion* (GBM), *Generalized Autoregressive Conditional Heteroskedastic* (GARCH), *Fractional Brownian Motion* (FBM) and *Autoregressive Fractionally Integrated Moving Average* (ARFIMA) series, are reported in Section 3. Finally, results are discussed, conclusions are drawn and a path for future work is suggested in Section 4.

## 2. Methods and Data

In this section the cluster entropy approach developed in [9,10] is briefly recalled. The second part of this section is devoted to the description of financial market data used in [23]. For the sake of completeness, we also recall the main definitions related to the Geometric Brownian Motion, Generalized Autoregressive Conditional Heteroskedastic, Fractional Brownian Motion, Autoregressive Fractionally Integrated Moving Average processes.

### 2.1. Cluster Entropy Method

It is well-known that the general idea behind Shannon entropy is to measure the amount of information embedded in a message to identify the shortest subsequence actually carrying the relevant information and the degree of redundancy which is not necessary to reproduce the initial message. The Shannon functional is written as:

(1) S(τ,n)=∑P(τ,n)logP(τ,n),

where P(τ,n) is a probability distribution associated with the time series y(t). To estimate the probability distribution P(τ,n), it is necessary to partition the continuous phase space into disjoints sets. The traditionally adopted methods divide the sequence into segments of equal lengths (blocks). Here, we follow another approach.

In [9,10] the time sequence y(t), is partitioned in *clusters* by the intersection with its moving average ${\tilde{y}}_{n}\left(t\right)$, with *n* the size of the moving average. The simplest type of moving average is defined at each *t* as the average of the *n* past observation from *t* to t−n+1,

(2) $${\tilde{y}}_{n}\left(t\right)=\frac{1}{n}\sum_{k=0}^{n-1}y\left(t-k\right).$$

Note that while the original series is defined from 1 to *N*, the moving average series is defined from 1 to N−n+1 because *n* samples are necessary to initialize the series. The original series and the moving average series are indicated as ${\left\{y\left(t\right)\right\}}_{t=1}^{N}$ and ${\left\{{\tilde{y}}_{n}\left(t\right)\right\}}_{t=1}^{N-n+1}$ respectively. Consecutive intersections of the time series and of the moving average series yield a partition of the phase space into a series of *clusters*. Each cluster is defined as the portion of the time series y(t) between two consecutive intersection of y(t) itself and its moving average ${\tilde{y}}_{n}\left(t\right)$ and has length (or duration) equal to:

(3) $$\tau_{j}\equiv \left|\right|t_{j}-t_{j-1}\left|\right|,$$

where $t_{j-1}$ and $t_{j}$ refers to two subsequent intersections of y(t) and ${\tilde{y}}_{n}\left(t\right)$. For each moving average window *n*, the probability distribution function P(τ,n), i.e., the frequency of the cluster lengths τ, can be obtained by counting the number of clusters $N_{j}\left(\tau_{j},n\right)$ with length $\tau_{j}$, j∈{1,N−n−1}. The probability distribution function P(τ,n) results:

(4) $$P\left(\tau ,n\right)∼\tau^{-D}F\left(\tau ,n\right)\;,$$

where the exponent *D* indicates the fractal dimension and can be expressed as

(5) D=2−H

with *H* the Hurst exponent of the sequence. Hence, the fractal dimension ranges between 1<D<2, as the Hurst exponent varies between 0<H<1. In this framework long-range correlation implies that the clusters are organized in a similar way along the time series (self-organized), even for clusters far away in time from each other. The term F(τ,n) in Equation (4) takes the form:

(6) $$F\left(\tau ,n\right)\equiv e^{-\tau /n}\;,$$

to account for the drop-off of the power-law behavior for τ<n and the onset of the exponential decay when τ≥n due to the finiteness of *n*. When n→1 the lengths τ of clusters tend to be centered around a single value. When n→N, that is when *n* tends to the length of the whole sequence, only one cluster with τ=N is generated. For middle values of *n* however a broader range of lengths is obtained and therefore the probability distribution spreads all values. When the probability distribution in Equation (4) is fed into the Shannon functional in Equation (1) the result is the following:

(7) $$S\left(\tau ,n\right)=S_{0}+\log\tau^{D}-\log F\left(\tau ,n\right),$$

which, after substituting Equation (6), becomes:

(8) $$S\left(\tau ,n\right)=S_{0}+\log\tau^{D}+\frac{\tau }{n},$$

where $S_{0}$ is a constant, $\log\tau^{D}$ accounts for power-law correlated clusters related to $\tau^{-D}$ and τ/n accounts for exponentially correlated clusters related to the term F(τ,n). The term $S_{0}$ can be evaluated in the limit τ∼n→1, which results in $S_{0}\to -1$ and S(τ,n)→0, that corresponds to the fully deterministic case, where each cluster has size equal to 1. On the other hand, when τ∼n→N, the maximum value for the entropy is obtained with $S\left(\tau ,n\right)=\log N^{D}$, which corresponds to the case of maximum randomness, where there is one cluster coinciding with the whole series. Equation (8) shows that power-law correlated clusters, characterized by having length τ<n, are described by a logarithmic term as $\log\tau^{D}$, and their entropy do not depend on the moving average window *n*. However, for values of τ≥n, which represent exponentially correlated clusters, the term τ/n becomes predominant. Cluster entropy increases linearly as τ/n, with slope decreasing as 1/n. Hence, due to the finite size effects introduced by the partitioning method, in τ=n the behavior of entropy changes and its values exceeds the curve $\log\tau^{D}$. In other words, clusters that are power-law correlated does not depend on *n*, are said to be *ordered* and represent deterministic information. Clusters that are exponentially correlated does depend on *n*, are said to be *disordered* and represent random clusters.

The meaning of entropy in information theory can be related to the corresponding concepts in thermodynamics. In an *isolated system*, the entropy increase dS refers to the irreversible processes spontaneously occurring within the system. In an *open system*, an additional entropy increase $dS_{ext}$ should be taken into account due to the interaction with the external environment.

The term $\log\tau^{D}$ should be interpreted as the entropy of the isolated system. It is independent on *n*, that is it is independent on the partitioning method. It takes the form of the Boltzmann entropy, that can be written as S=logΩ, with Ω the volume of the system. Therefore the quantity $\tau^{D}$ corresponds to the volume occupied by the fractional random walker.

The term τ/n represents the excess entropy caused by the external process of partitioning the sequence. The excess entropy depends on the moving average window *n*. If same size boxes were chosen, the excess entropy term τ/n would vanish and entropy would reduce to the logarithmic term. When a moving average partition is used, the term τ/n emerges to account for the additional heterogeneity introduced by the randomness of the process. Thence, for exponentially correlated clusters entropy exceeds the logarithmic asymptotic.

In order to increase the sensitivity of the method, the integral of the entropy function over the clusters length τ can be considered:

(9) I(n)=∫S(τ,n)dτ,

which for discrete sets reduces to $I\left(n\right)=\sum_{\tau }S\left(\tau ,n\right)$. The function I(n) is a cumulative entropy measure able to embed all the information in a single figure.

Equation (9) can be written as:

(10) $$I\left(n\right)=\int_{1}^{\tau (n)}S\left(\tau ,n\right)d\tau +\int_{\tau (n)}^{\infty }S\left(\tau ,n\right)d\tau \;.$$

The first integration is referred to the power law regime of the cluster entropy, the second integration is referred to the linear regime of the cluster entropy (i.e., the excess entropy term).

### 2.2. Financial Data

The objective of this work is to investigate and shed light on the characteristic features exhibited by cluster entropy of financial markets. In particular here our focus is on the systematic dependence of the cluster entropy of the price series over time horizon *M*.

In [23] the cluster entropy is applied to a large set of tick-by-tick data of the USA’s indexes (S&P500, NASDAQ and DJIA). NASDAQ is an index resulting from all the public firms quoted on the market, DJIA and S&P500 indexes are representative of a selected number of public firms. For each index, investigated data include tick-by-tick prices from January 2018 to December 2018. As the main goal of the paper is to quantify the intrinsic dynamics of prices and to capture the endogenous sources of risk over different temporal horizons, a year of data with no external shocks or crisis have been chosen. More information about the markets can be found at the Bloomberg terminal.

To study the dynamics of financial series different time horizons need to be compared. As explained in the Introduction, entropy is sample-size dependent by definition, thus in order to rule out spurious results the length of the investigated sequences must be the same. Therefore, cluster entropy analysis requires the comparison to be implemented on series with same length. Raw data have been downloaded from the Bloomberg terminal in the form of tick-by-tick data. The lengths of the raw series vary due to different number of trading days and transactions per time unit. It is therefore necessary, as first computational step, to implement a sampling of the raw data to make the length of the series exactly the same. The first raw series ranges from the first transaction of January 2018 to the last one of January 2018; the second ranges from the first transaction in January 2018 to the last of February 2018, *…*, the twelfth ranges from the first transaction in January 2018 to the last of December 2018, a period equivalent to the whole year. Because each raw series ranges from the first tick of 2018 to the last tick of the relative month, the twelve series have very different lengths. The series are sampled to obtain twelve *series* with same length as described in the following.

Twelve sampling time intervals and corresponding frequencies must be defined, i.e., twelve integers indicating for each series the interval of skipped data. Sampling intervals are obtained by dividing the length of each raw series by the length of the shortest raw one and then rounding to the inferior integer. Thence, each raw series is sampled with the relative sampling interval to yield a *sampled series*: for each sample in the sampled series, a number of samples equal to the sampling frequency has been discarded in the raw series. The sampled series obtained are *approximately* of equal lengths. To obtain twelve series of *exactly* equal length, a few observations are cut off, when exceeding the length of the shortest series. The result consists in twelve sampled series that are equal in length and refer to time horizons varying from one month (M=1) to twelve months (M=12). In more details, $N_{M}$ is the length of the series corresponding to the horizon *M* (where *M* ranges from 1 to 12 for one year of data). The shortest monthly series is used to evaluate the minimum value $N_{M}^{*}$ and the corresponding sampling frequency. Then, the sampling intervals for the multiple periods is derived by dividing the multiple period lengths (i.e., the sum of multiple consecutive $N_{M}$) by the value $N_{M}^{*}$. In Table 1 a few examples of sampling intervals and lengths $N_{M}$ are shown to clarify the procedure. It is worth noting that the length of sampled series should be at least $10^{5}$ to ensure enough accuracy of the results.

### 2.3. Artificial Data

Artificial series have been generated by using Geometric Brownian Motion, Generalized Autoregressive Conditional Heteroskedastic, Fractional Brownian Motion and Autoregressive Fractionally Integrated Moving Average processes with same temporal structure corresponding to the different horizons of the financial market data reported in [23]. Then the sampling method proceeds analogously from the calculation of the sampling frequency. Such sampling method was applied to series generated by artificial financial models to make sure that the information content would be comparable to that of real-world financial series. In the remainder of this section, we recall the main definitions for the afore mentioned processes.

#### 2.3.1. Geometric Brownian Motion

The Geometric Brownian Motion is the basis of the *Black-Scholes-Merton* model used to price options and is defined by the following difference equation:

(11) $$dX_{t}=\mu \left(t\right)X_{t}dt+D\left(t,X_{t}\right)\sigma \left(t\right)dB_{t},$$

where μ(t) indicates the level of return, σ(t) the volatility and $dB_{t}$ is a simple Brownian motion. Volatility is deterministic and constant and there are no jumps. Increments are independent on previous states.

#### 2.3.2. Autoregressive Conditional Heteroskedasticity Models

We perform simulations by using GARCH(1,1) of the broad family of the autoregressive conditional heteroscedasticity (ARCH) models. It describes the variance of the current error term or innovation as a function of previous values. The GARCH(1,1) model is defined by the following relationships:

$$\begin{array}{c} \mu \left(t\right)-E_{t-1}\mu \left(t\right)=\sqrt{\sigma_{t}}\varepsilon_{t}\\ \sigma_{t}=\omega +\alpha \varepsilon_{t-1}^{2}+\beta \sigma_{t-1} \end{array}$$

where μ(t) represents the return of an asset at time *t*, $E_{t-1}\left(\mu_{t}\right)$ is the expected return at $t-1,\sigma_{t}$ characterises the conditional volatility at time t, and $\varepsilon_{t}$ is the innovation term at time *t*.

#### 2.3.3. Fractional Brownian Motion

The *Fractional Brownian Motion* is a long memory process introduced in [34]:

(12) $$\begin{array}{ll} B_{H}\left(t\right)=B_{H}\left(0\right) & +\frac{1}{\Gamma (H+1/2)}(\int_{-\infty }^{0}({\left(t-s\right)}^{H-1/2}\\ & -{\left(-s\right)}^{H-1/2})dB\left(s\right)+\int_{0}^{t}{\left(t-s\right)}^{H-1/2}dB\left(s\right)). \end{array}$$

It is also referred to as a *self-similar* process. A stochastic process $X_{t}$, with t∈R, is said to be self-similar if there exist H>0 such that for any *scaling factor*c>0,

(13) $$X_{ct}\overset{L}{=}c^{H}X_{t},$$

with *H* the Hurst exponent and ($\overset{L}{=}$) equivalence in distribution. Self-similar processes are stochastic models where a scaling in time is equivalent, *in term of distribution*, to an appropriate scaling in space. Moreover, if, for any *k*, the distribution of $\left(X_{t_{1}+c}-X_{t_{1}+c-1},...,X_{t_{k}+c}-X_{t_{k}+c-1}\right)$ does not depend on *c*, $X_{t}$ is said to be self-similar with *stationary increments*. So, a Gaussian process $B_{H}\left(t\right)$ is called a *Fractional Brownian Motion*, if it satisfies: 1. $B_{H}\left(t\right)$ is self-similar with 0<H<1; 2. $B_{H}\left(t\right)$ has stationary increments. When H=0.5 a simple Brownian Motion with independent increments is recovered. When 0<H<0.5 the *Fractional Brownian Motion* is said to be anti-persistent, which means that increments tend to be opposite signed. Conversely, when 0.5<H<1 it is said to be persistent, which means that increments tend to be equally signed.

#### 2.3.4. Autoregressive Fractionally Integrated Moving Average

The *Autoregressive Fractionally Integrated Moving Average* (*ARFIMA*) is one of the most common processes to model long-range correlated asset prices. The Autoregressive Fractionally Integrated Moving Average process of order (p,d,q) with mean μ, may be written, using the lag operator *L*, as:

(14) $$\Phi \left(L\right){\left(1-L\right)}^{d}\left(y_{t}-\mu \right)=\Theta \left(L\right)\epsilon_{t},$$

with $\epsilon_{t}\;i.i.d.$ and $∼(0,\sigma_{\epsilon }^{2})$. The autoregressive component of the process is represented by the factor:

(15) $$\Phi \left(L\right)=1-\phi_{1}L-...-\phi_{p}L^{p},$$

where the lag operator of order *p* shifts the value of $y_{t}$ back to *p* observations, so that one obtains:

(16) $$\Phi \left(L\right)y_{t}=\left(1-\phi_{1}L-...-\phi_{p}L^{p}\right)y_{t}=y_{t}-\phi_{1}y_{t-1}-\ldots -\phi_{p}y_{t-p}.$$

The moving average component of the process is represented by the factor:

(17) $$\Theta \left(L\right)\epsilon_{t}=\left(1+\theta_{1}L+\ldots +\theta_{q}L^{q}\right)\epsilon_{t}=\epsilon_{t}+\theta_{1}\epsilon_{t-1}+\ldots +\theta_{q}\epsilon_{t-q}\;.$$

The fractionally differencing operator ${\left(1-L\right)}^{d}$ is defined as:

(18) $${\left(1-L\right)}^{d}=\sum_{n=0}^{\infty }\frac{\Gamma \left(k-d\right)L^{k}}{\Gamma (-d)\Gamma (k+1)}.$$

Note that the process is stationary only for −0.5<d<0.5. For d<|0.5| the ARFIMA process is said to exhibit long memory.

The power spectral representation f(λ) of Fractional Brownian Motions and Autoregressive Fractionally Integrated Moving Average Processes provides further details regarding their power law behavior and the relation between the characteristic exponents. It is:

(19) $$\begin{array}{cc} f(\lambda ) & ∼{\left|\lambda \right|}^{-2d}\;\;\left(\mathrm{ARFIMA}\right)\\ f(\lambda ) & ∼{\left|\lambda \right|}^{1-2H}\;\;\left(\mathrm{FBM}\right) \end{array}$$

yielding:

(20) H=d+1/2.

## 3. Results

In this section, the results of the application of the cluster entropy method to several FBM and ARFIMA series are presented. The moving average cluster entropy can be implemented via the MATLAB codes available at [35].

**Figure 1:**
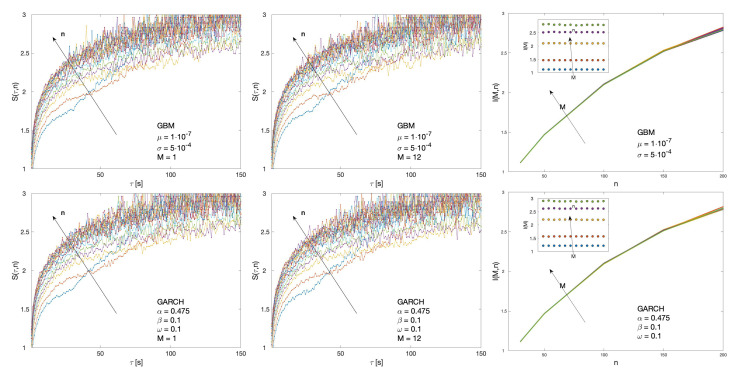
Cluster entropy results for Geometric Brownian Motion (GBM) and Generalized Autoregressive Conditional Heteroskedastic (GARCH) series are reported respectively in the first and in the second row. GBM series are generated with following parameters: $\mu =1\times 10^{-7}$ and $\sigma =5\times 10^{-4}$. GARCH series are generated with the following parameters: α=0.475, β=0.1 and ω=0.1. Left and middle panels show cluster entropy curves for time horizons M=1 and M=12. Right panels show Market Dynamic Index I(M,n) for different horizons *M* and moving average windows *n*. I(M,n) is independent on *M*.

First, a set of benchmark values for the cluster entropy are obtained by implementing the algorithm on Geometric Brownian Motion (GBM) and Generalized Autoregressive Conditional Heteroskedastic (GARCH) series. Geometric Brownian Motion series are generated by means of the MATLAB tool available at [36]. GBM series are analysed with parameters varying in the range $0\leq \mu \leq 1\times 10^{-7}$ and $5\times 10^{-4}\leq \sigma \leq 5\times 10^{-6}$. GARCH series are generated by using the computational tool provided in MATLAB [37]. Figure 1 reports cluster entropy and market dynamic index results obtained on GBM and GARCH series. The GBM series are generated with the following parameters: $\mu =1\times 10^{-7}$ and $\sigma =5\times 10^{-4}$; the GARCH series are generated with the following parameters: α=0.475, β=0.1 and ω=0.1. Left and middle panels show cluster entropy curves for time horizons M=1 and M=12, i.e., corresponding respectively to one period (one month) and twelve periods (one year) of data. Right panels show Market Dynamic Index I(M,n) for different horizons *M* and moving average windows *n*. I(M,n) does not depend on the temporal horizon *M* both in GBM and GARCH series.

**Figure 2:**
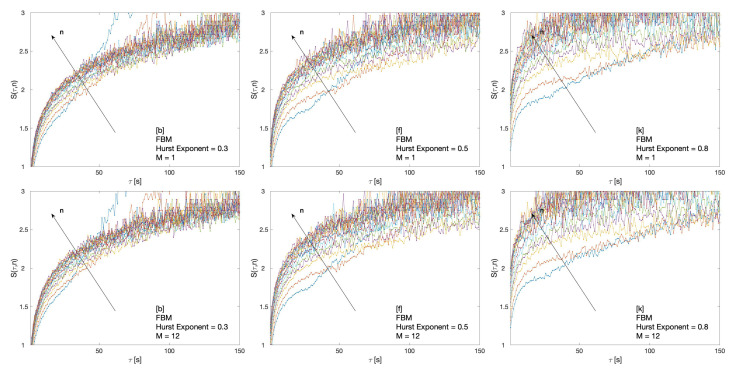
Cluster entropy results for Fractional Brownian Motion (FBM) series with H=0.3, H=0.5, H=0.8. First row shows results for time horizon M=1 (approximately equivalent to the first month (January 2018) of raw data for NASDAQ, S&P500, DIJA). The second row shows results for time horizon M=12 (approximately equivalent to twelve months of data in NASDAQ, S&P500, DIJA, i.e, the whole 2018 year).

Results of the cluster entropy approach applied to Fractional Brownian Motion (FBM) are reported in Figure 2. The Fractional Brownian Motion series were generated by means of the FRACLAB tool available at [38]. Several Fractional Brownian Motion series with Hurst exponent varying in the range 0.1≤H≤0.9 are analysed. Figure 2 shows the cluster entropy for time horizon M=1 and M=12 for FBM series with H=0.3, H=0.5 and H=0.8.

In general, cluster entropy calculated at different time horizons *M* presents a similar behavior. On account of Equation (8), power-law correlated clusters with a smooth logarithmic increase of the entropy for τ<n can be expected. Conversely, for τ≥n, the exponentially correlated decay sets the entropy to increase linearly with the term τ/n dominating. However, a quite different behavior is observed for different *H*. For H=0.3 (anti-correlated FBM series), cluster entropy curves exhibit a very limited dependence on the moving average window *n* over the range of investigated τ. For H=0.5, cluster entropy curves vary more significantly as the moving average window *n* changes. For H=0.8, cluster entropy curves vary even more remarkably by taking higher values for increasing *n*.

**Figure 3:**
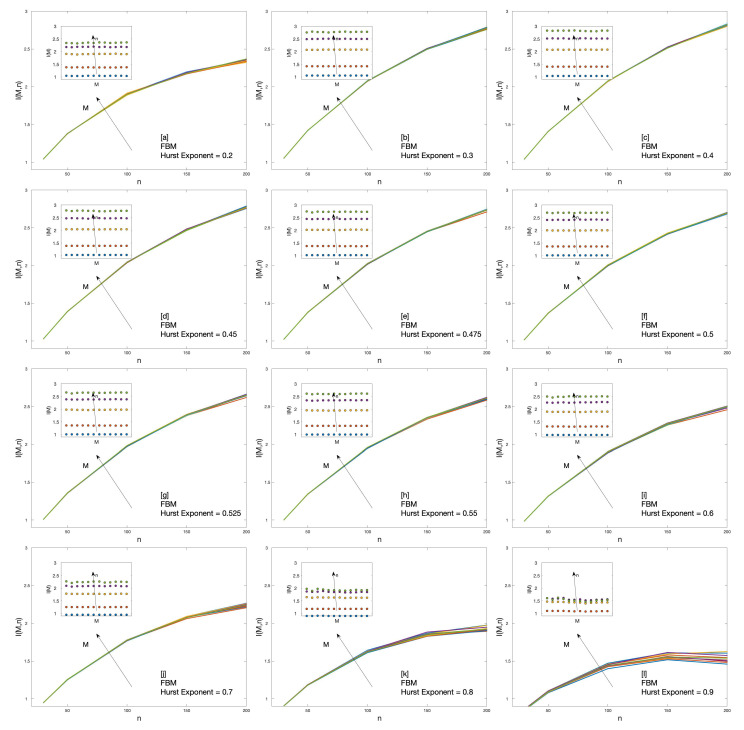
Market Dynamic Index I(M,n) for Fractional Brownian Motion series with Hurst exponent ranging from H=0.2 to H=0.9 respectively from (a) to (l).

The dependence of the cluster entropy on temporal horizon *M* is reflected in the results of the Market Dynamic Index I(M,n) plotted in Figure 3. The Market Dynamic Index I(M,n) is estimated over several FBM series with different Hurst exponents *H*. For anticorrelated series 0≤H≤0.5, I(M,n) curves overlap for all the moving average windows *n* and time horizons *M*. For positively correlated series 0.5≤H≤0.9, I(M,n) exhibits slightly different values as a function of time horizons *M*. It is worth-noting that the magnitude of the marginal increments in I(M,n) at large *n* increases as *H* increases for 0≤H≤0.5, reaches a maximum for H=0.5 and then decreases again for 0.5≤H≤0.9. This effect is evident in the insets of Figure 3.

The cluster entropy analysis is implemented on *Autoregressive Fractionally Integrated Moving Average* (ARFIMA) series obtained by means of simulations for several combination of parameters [39]. The extent of investigated parameters are marked by alphabet labels in Table 2 for ARFIMA (1,d,1) and in Table 3 for ARFIMA (3,d,2) and ARFIMA(1,d,3).

**Figure 4:**
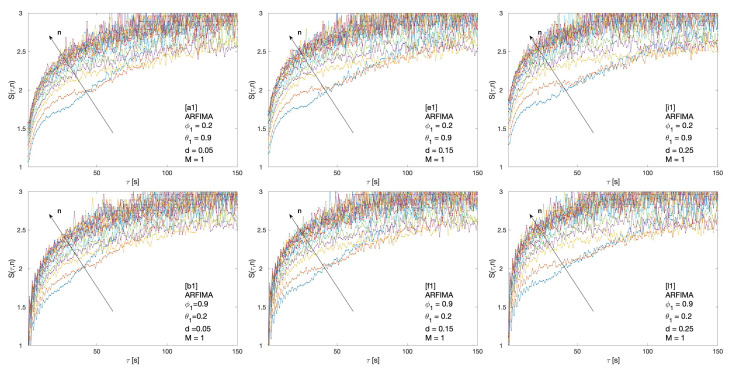
Cluster entropy results for horizon M=1 for ARFIMA series with different combinations of the differencing parameter *d*, autoregressive parameter ϕ and moving average parameter θ. The differencing parameter takes values d=0.05, d=0.15, d=0.25 with a different combinations of autoregressive and moving average parameter. The full set of analysed values of *d*, ϕ and θ is reported in Table 2.

**Figure 5:**
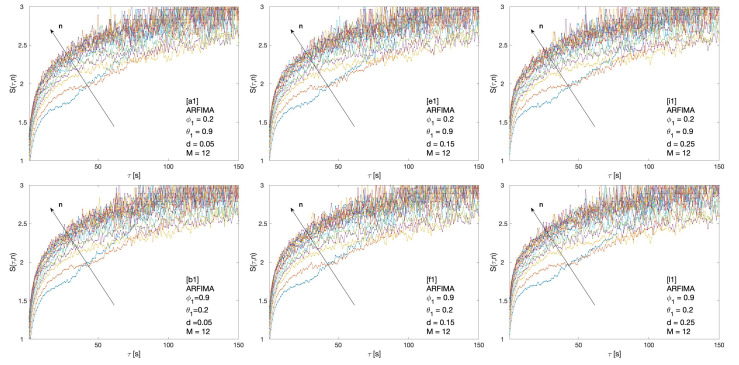
Cluster entropy results for horizon M=12 on ARFIMA series with different combinations of the differencing parameter *d*, autoregressive parameter ϕ and moving average parameter θ. The differencing parameter takes values d=0.05, d=0.15 and d=0.25 with a different combination of autoregressive and moving average parameters. The full set of analysed values of *d*, ϕ and θ is reported in Table 2.

**Figure 6:**
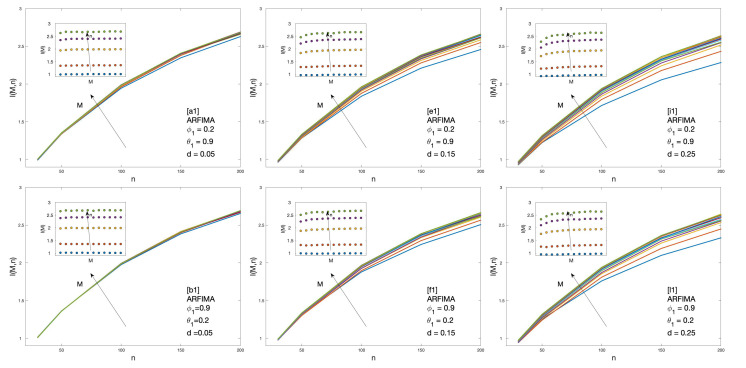
Market Dynamic Index I(M,n) for ARFIMA series with different combinations of the differencing parameter *d*, autoregressive parameter ϕ, and moving average parameter θ. The differencing parameter takes values d=0.05, d=0.15, d=0.25, with a different combination of autoregressive and moving average parameters. The full set of analysed values of *d*, ϕ and θ is reported in Table 2.

**Figure 7:**
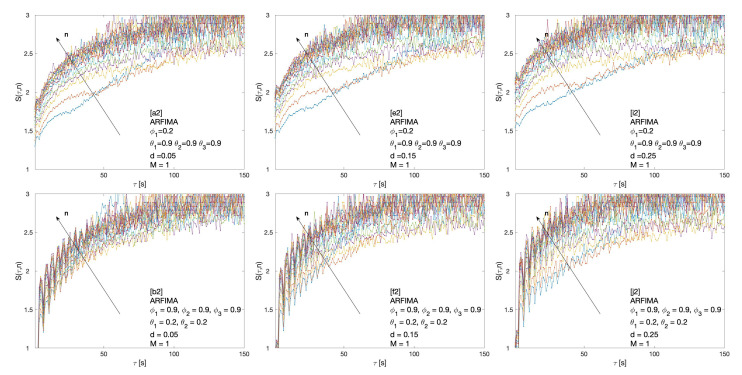
Cluster entropy results for horizon M=1 on ARFIMA series with different combinations of the differencing parameter *d*, autoregressive parameter $\phi_{1}$, $\phi_{2}$, and $\phi_{3}$ and moving average parameter $\theta_{1}$, $\theta_{2}$ and $\theta_{3}$. The differencing parameter takes values d=0.05, d=0.15, d=0.25, with a different combination of autoregressive and moving average parameters. The full set of analysed values of *d*, ϕ and θ is reported in Table 3.

**Figure 8:**
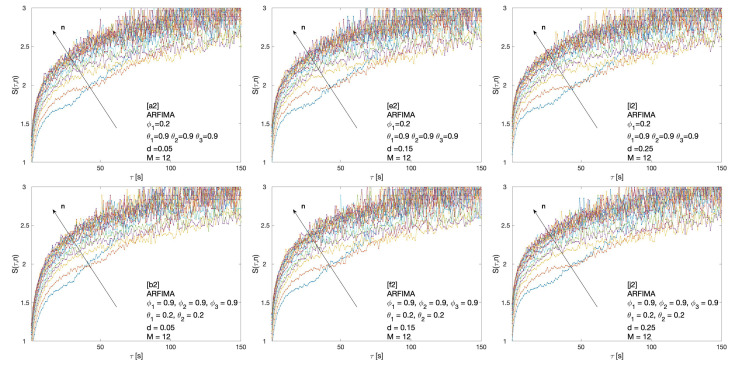
Cluster entropy results for horizon M=12 on ARFIMA series with different combinations of the differencing parameter *d*, autoregressive parameter $\phi_{1}$, $\phi_{2}$, and $\phi_{3}$ and moving average parameter $\theta_{1}$, $\theta_{2}$ and $\theta_{3}$. The differencing parameter takes values d=0.05, d=0.15 and d=0.25, with a different combination of autoregressive and moving average parameters. The full set of analysed values of *d*, ϕ and θ is reported in Table 3.

**Figure 9:**
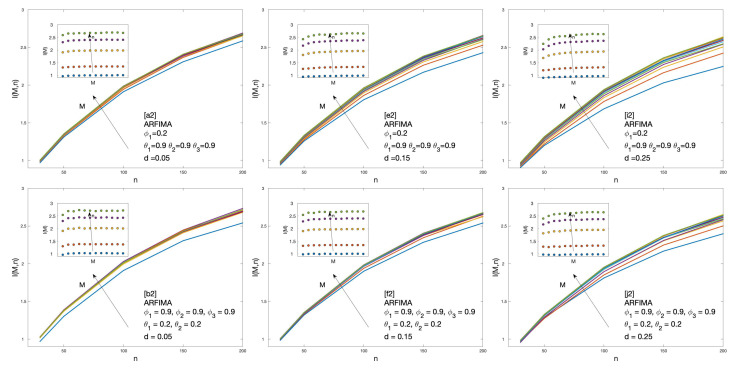
Market Dynamic Index I(M,n) for ARFIMA series with different combinations of the differencing parameter *d*, autoregressive parameter $\phi_{1}$, $\phi_{2}$, and $\phi_{3}$ and moving average parameter $\theta_{1}$, $\theta_{2}$ and $\theta_{3}$. The differencing parameter takes values d=0.05, d=0.15 and d=0.25, with a different combination of autoregressive and moving average parameters as reported in Table 3.

Cluster entropy results for ARFIMA (1,d,1), corresponding to parameters marked by alphabet labels in Table 2, are shown in Figure 4 and Figure 5. The corresponding market dynamic indexes I(M,n) calculated by using the data of the cluster entropy results on ARFIMA (1,d,1) are shown in Figure 6. Cluster entropy results on ARFIMA (3,d,2) and ARFIMA(1,d,3), corresponding to parameters marked by alphabet labels in Table 3, are shown in Figure 7 and Figure 8. Market Dynamic Index for series generated by ARFIMA (3,d,2) and ARFIMA(1,d,3) processes are reported in Figure 9. With differencing parameter 0<d<0.2, Market Dynamic Index curves are *n*-invariant for small values of *n*, but horizon dependence emerges at larger *n*. When 0.2<d<0.5, Market Dynamic Index curves show a significant horizon dependence even at small *n*. Therefore, according to the choice of the differencing parameter *d*, series generated by ARFIMA processes can reproduce the effect shown by the cluster entropy in real-world financial markets.

## 4. Discussion and Conclusions

The cluster entropy behavior described by Equation (8) has been replicated by simulations performed on artificially generated series, with results reported in Section 3. Figures show cluster entropy results for the following processes: Geometric Brownian Motion (Figure 1); Generalized Autoregressive Conditional Heteroskedastic processes (Figure 1); Fractional Brownian Motion (Figure 2); Autoregressive Fractionally Integrated Processes (Figure 4, Figure 5, Figure 7 and Figure 8). The focus here is limited to the results shown in Figure 2, Figure 4, Figure 5, Figure 7 and Figure 8 related to FBM and ARFIMA because they are long-range dependent models relevant to the present analysis. The behavior of cluster entropy curves is well represented by Equation (8), while deviations occur in *extreme cases*, as in the case of ARFIMA(5,d,0) models generated by autoregressive parameters $\theta_{i}≃0.9$, that are far away from those observed in real markets. In general, power-law correlated clusters, characterized by length τ<n, determine the logarithmic behavior and the entropy term $\log\tau^{D}$, regardless of the moving average window value *n*. On the other hand, exponentially correlated clusters, characterized by length τ>n, are related to the linear behavior prescribed by the excess entropy term τ/n, which depends on the moving average window *n*.

Cumulative measures are useful to summarize key information in a single figure. Thus, the Market Dynamic Index I(M,n) is deduced from the cluster entropy results by means of Equation (10). I(M,n) gathers the information present in the FBM series at different time horizons *M* and moving average windows *n* as shown in Figure 3. The Market Dynamic Index I(M,n) replicates the characteristic behaviour observed in real world financial markets [23] when estimated in long-range positively correlated sequences. Conversely, one can note that the Market Dynamic Index I(M,n) for Fractional Brownian processes with Hurst exponent 0<H<0.5 (anticorrelated FBMs) does not present any horizon dependence. Conversely, Fractional Brownian Motion series with 0.5<H<1 (positively correlated FBMs) do show horizon dependence. However, as it will be discussed below, Fractional Brownian Motion series fail to fully reproduce the financial markets behavior.

In the case of the ARFIMA processes, a significant horizon dependence emerges, as observed in the Market Dynamic Index curves plotted in Figure 6 and Figure 9. Thus, cluster entropy for ARFIMA processes exhibits horizon dependence as observed in real world financial markets. The extent of long range dependence and its microscopic origin are consistent with findings of previous studies [27,28].

To further validate the findings, statistical significance has been checked by using the *T-paired test* of the null hypothesis $h_{0}$ that the cluster entropy values obtained by ARFIMA simulations come from distributions with equal mean, variance and probability *p* as the simple Brownian Motion (H=0.5), assumed as benchmark. The results of *T-paired test* are reported in Table 4 (for the sake of comparison the results of the *T-paired test* performed on NASDAQ, DJIA and S&P500 markets in Table 5 [23] are also included here).

A qualitative comparison between Table 4 and Table 5 suggests an overall similarity between ARFIMA and real world markets behaviour. In particular, *p* values in column *[f1]* are quite close to those of the S&P500 suggesting a correlation degree with Hurst exponent H≃0.65 and differencing parameter d≃0.15 for S&P500. Probability values *p* in column *[e2]* are also close to S&P500, confirming the value H≃0.65 and d≃0.15. The probability values for DJIA are better approximated by the set of ARFIMA parameters in column *[b1]* and column *[a2]* suggesting lower values of the correlation exponents: H≃0.55 and d≃0.05. The lower values of the probability *p* indicate a more complex behavior of the NASDAQ with stronger deviation from the fully uncorrelated Brownian motion. By looking at Table 4, one can relate the NASDAQ behaviour to higher values of the long-range parameters of the ARFIMA model. In particular, the NASDAQ probability values become closer to parameter sets *[i2]* and *[n2]* corresponding to higher correlation degrees and correlation exponents around H≃0.75 and d≃0.25. The different horizon dependence of NASDAQ and DJIA, where the former is a diversified stock market with a high degree of heterogeneity and the latter is an index representative of a chosen set of industrial stocks, is consistent with the ability of the cluster entropy index to quantify market heterogeneity.

The cluster entropy behavior appears deeply related to positive persistence and long-range correlation. In real-world financial series, horizon dependence deviates from the behaviour of fully uncorrelated series. The Market Dynamic Index, obtained via the integration of the cluster entropy curves, provides this feature in a cumulative, thus more robust, form. In conclusion, contrary to the assumptions of the traditional financial market theories, the hypothesis of efficient markets and rational investor behavior do not hold on account of the horizon dependence of the cluster entropy.

## Figures and Tables

**Table 1:** Example of lengths and time horizon *M* for NASDAQ data (2018). The 2nd column corresponds to the number of transactions over the horizon *M*. These lengths are used as a reference to generate artificial series to allow a direct comparison between results obtained on real and artificial data. The 3rd column corresponds to the sampled lengths used in the calculation of the cluster entropy. The 4th and 5th columns correspond respectively to the raw and rounded time intervals obtained dividing $N_{M}$ by the series of shortest length $N_{M}^{*}$.

*M*	$N_{M}$	$N_{M}^{*}$	$t_{S}$	$t_{S}^{*}$
1	586,866	586,866	1.0000	1
2	1,117,840	586,866	1.9048	1
3	1,704,706	586,866	2.9048	2
4	2,291,572	586,866	3.9048	3
5	2,906,384	586,866	4.9524	4
6	3,493,250	586,866	5.9524	5
7	4,069,315	586,866	6.9340	6
8	4,712,062	586,866	8.0292	8
9	5,243,029	586,866	8.9339	8
10	5,885,781	586,866	10.0292	10
11	6,461,845	586,866	11.0108	11
12	6,982,017	586,866	11.8971	11

**Table 2:** Full set of parameter range for the ARFIMA (1,d,1) processes simulated in this work. Specifically, *D* is the fractal dimension, *H* is the Hurst exponent and *d* is the differencing parameter (1st, 2nd and 3rd columns) which are related by Equation (20), ϕ is the autoregressive parameter (4th column), and θ is the moving average parameter (5th column). *Label* refers to each parameter set (6th column). Specifically, cluster entropy results for the parameter sets: [a1], [b1], [e1], [f1], [i1], [l1] are plotted in Figure 4 (M = 1) and Figure 5 (M = 12), while the Market Dynamic Index is plotted in Figure 6.

*D*	*H*	*d*	ϕ	θ	Label
1.45	0.55	0.05	0.20	0.90	a1
			0.90	0.20	b1
1.40	0.60	0.10	0.20	0.90	c1
			0.90	0.20	d1
1.35	0.65	0.15	0.20	0.90	e1
			0.90	0.20	f1
1.30	0.70	0.20	0.20	0.90	g1
			0.90	0.20	h1
1.25	0.75	0.25	0.20	0.90	i1
			0.30	0.40	j1
				0.85	k1
			0.90	0.20	l1
				0.40	m1
				0.85	n1
1.20	0.80	0.30	0.20	0.90	o1
			0.90	0.20	p1
1.02	0.98	0.48	0.30	0.40	q1
				0.85	r1
			0.90	0.40	s1
				0.85	t1

**Table 3:** Full set of parameter range for ARFIMA (3,d,2) and ARFIMA(1,d,3) processes simulated in this work. Specifically *H* is the Hurst exponent and *d* is the differencing parameter which are related by Equation (20) (1st and 2nd columns); $\phi_{1}$, $\phi_{2}$ and $\phi_{3}$ are the autoregressive parameters (3rd, 4th and 5th columns); $\theta_{1}$, $\theta_{2}$ and $\theta_{3}$ are the moving average parameters (6th, 7th and 8th columns). *Label* refers to each parameter set (10th column). Specifically, cluster entropy results for the parameter sets: [a2], [b2], [e2], [f2], [i2], [j2] are plotted in Figure 7 (M = 1) and Figure 8 (M = 12), while the Market Dynamic Index is plotted in Figure 9.

*D*	*H*	*d*	$\phi_{1}$	$\phi_{2}$	$\phi_{3}$	$\theta_{1}$	$\theta_{2}$	$\theta_{3}$	*Label*
1.45	0.55	0.05	0.20	-	-	0.90	0.90	0.90	a2
			0.90	0.90	0.90	0.20	0.20	-	b2
1.40	0.60	0.10	0.20	-	-	0.90	0.90	0.90	c2
			0.90	0.90	0.90	0.20	0.20	-	d2
1.35	0.65	0.15	0.20	-	-	0.90	0.90	0.90	e2
			0.90	0.90	0.90	0.20	0.20	-	f2
1.30	0.70	0.20	0.20	-	-	0.90	0.90	0.90	g2
			0.90	0.90	0.90	0.20	0.20	-	h2
1.25	0.75	0.25	0.20	-	-	0.90	0.90	0.90	i2
			0.90	0.90	0.90	0.20	0.20	-	j2
1.20	0.80	0.30	0.20	-	-	0.90	0.90	0.90	k2
			0.40	0.16	-	0.90	0.81	0.73	l2
			0.90	0.90	0.90	0.20	0.20	-	m2
1.15	0.85	0.35	0.20	-	-	0.90	0.90	0.90	n2
1.02	0.98	0.48	0.40	0.16	-	0.90	0.81	0.73	o2

**Table 4:** Probability *p* to reject the null hypothesis that the cluster entropy values for the ARFIMA processes at varying horizons *M*, have same mean and variance of the Fractional Brownian Motion with H=0.5. The probability *p* has been estimated by standard *T-paired test*. First column reports the temporal horizon *M*. The other columns refers to parameter sets *[b1]*, *[f1]*, *[l1]*, *[a2]*, *[e2]*, *[i2]*, *[n2]*, *[o2]* of Table 2 and Table 3.

*M*	*[b1]*	*[f1]*	*[l1]*	*[a2]*	*[e2]*	*[i2]*	*[n2]*	*[o2]*
1	0.9597	0.7938	0.6013	0.8519	0.6779	0.4956	0.3542	0.2314
2	0.9863	0.8429	0.6985	0.9293	0.7883	0.6566	0.5414	0.4304
3	0.9820	0.8789	0.7743	0.938	0.8346	0.7362	0.6468	0.5576
4	0.9848	0.8922	0.8031	0.956	0.8689	0.7827	0.7147	0.6380
5	0.9878	0.9062	0.8325	0.9608	0.8809	0.8102	0.7528	0.6911
6	0.9940	0.9197	0.8517	0.9724	0.9043	0.8417	0.7840	0.7322
7	0.9785	0.9186	0.8633	0.9617	0.9038	0.8521	0.8036	0.7614
8	0.9930	0.9321	0.8775	0.9762	0.9229	0.8710	0.8333	0.7931
9	0.9867	0.9370	0.8890	0.9737	0.9273	0.8809	0.8438	0.8100
10	0.9813	0.9333	0.8952	0.9710	0.9261	0.8880	0.8533	0.8195
11	0.9816	0.9436	0.9011	0.9749	0.9326	0.8965	0.8643	0.8342
12	0.9853	0.9451	0.9072	0.9741	0.9353	0.9019	0.8764	0.8508

**Table 5:** Probability *p* to reject the null hypothesis that the cluster entropy values for the NASDAQ, DJIA and S&P500 at varying horizons *M* have same mean and variance of the Fractional Brownian Motion with H=0.5. First column reports the temporal horizon *M*. The probability *p* has been estimated by standard *T-paired test* [23].

*M*	NASDAQ	S&P500	DJIA
1	0.5154	0.7399	0.8892
2	0.6026	0.8335	0.9257
3	0.6470	0.8588	0.9332
4	0.6631	0.8814	0.9283
5	0.6823	0.9018	0.9417
6	0.7124	0.9246	0.9534
7	0.7162	0.9224	0.9461
8	0.7288	0.9309	0.9618
9	0.7370	0.9479	0.9645

## References

[1] Grassberger P., Procaccia I. (1983). Characterization of strange attractors. Phys. Rev. Lett..

[2] Crutchfield J.P. (2012). Between order and chaos. Nat. Phys..

[3] Ormos M., Zibriczky D. (2014). Entropy-based financial asset pricing. PLoS ONE.

[4] Yang J. (2018). Information Theoretic Approaches in Economics. J. Econ. Surv..

[5] Ghosh A., Julliard C., Taylor A.P. (2017). What Is the Consumption-CAPM Missing? An Information-Theoretic Framework for the Analysis of Asset Pricing Models. Rev. Financ. Stud..

[6] Backus D., Chernov M., Zin S. (2014). Sources of entropy in representative agent models. J. Financ..

[7] Zhou R., Cai R., Tong G. (2013). Applications of entropy in finance: A review. Entropy.

[8] Shalizi C.R., Shalizi K.L., Haslinger R. (2004). Quantifying self-organization with optimal predictors. Phys. Rev. Lett..

[9] Carbone A., Castelli G., Stanley H.E. (2004). Analysis of clusters formed by the moving average of a long-range correlated time series. Phys. Rev. E.

[10] Carbone A., Stanley H.E. (2007). Scaling properties and entropy of long-range correlated time series. Phys. A.

[11] Carbone A. (2013). Information Measure for Long-Range Correlated Sequences: The Case of the 24 Human Chromosomes. Sci. Rep..

[12] Zhao X., Sun Y., Li X., Shang P. (2018). Multiscale transfer entropy: Measuring information transfer on multiple time scales. Commun. Nonlinear Sci. Numer. Simul..

[13] Humeau-Heurtier A. (2015). The multiscale entropy algorithm and its variants: A review. Entropy.

[14] Niu H., Wang J. (2015). Quantifying complexity of financial short-term time series by composite multiscale entropy measure. Commun. Nonlinear Sci. Numer. Simul..

[15] Shannon C.E. (1948). A mathematical theory of communication, Part I, Part II. Bell Syst. Tech. J..

[16] Kolmogorov A.N. (1965). Three approaches to the quantitative definition ofinformation’. Probl. Inf. Transm..

[17] Li M., Vitányi P. (2008). An Introduction to Kolmogorov Complexity and Its Applications.

[18] Marcon E., Scotti I., Hérault B., Rossi V., Lang G. (2014). Generalization of the partitioning of Shannon diversity. PLoS ONE.

[19] Rubido N., Grebogi C., Baptista M.S. (2018). Entropy-based generating Markov partitions for complex systems. Chaos.

[20] Darbellay G.A., Vajda I. (1999). Estimation of the information by an adaptive partitioning of the observation space. IEEE Trans. Inf. Theory.

[21] Steuer R., Molgedey L., Ebeling W., Jimenez-Montaño M.A. (2001). Entropy and optimal partition for data analysis. Eur. Phys. J. B.

[22] Ponta L., Carbone A. (2018). Information measure for financial time series: Quantifying short-term market heterogeneity. Phys. A.

[23] Ponta L., Murialdo P., Carbone A. (2019). Quantifying horizon dependence of asset prices: A cluster entropy approach. arXiv.

[24] Vera-Valdés J.E. (2020). On Long Memory Origins and Forecast Horizons. J. Forecast..

[25] Graves T., Franzke C.L., Watkins N.W., Gramacy R.B., Tindale E. (2017). Systematic inference of the long-range dependence and heavy-tail distribution parameters of ARFIMA models. Phys. A.

[26] Bhattacharyya R., Datta R.P. (2020). The Dynamics of India’s Major Exchange Rates. Glob. Bus. Rev..

[27] Bhardwaj G., Swanson N. (2006). An empirical investigation of the usefulness of ARFIMA models for predicting macroeconomic and financial time series. J. Econom..

[28] Baillie R.T., Kongcharoen C., Kapetanios G. (2012). Prediction from ARFIMA models: Comparisons between MLE and semiparametric estimation procedures. Int. J. Forecast..

[29] Engle R.F., Rangel J.G. (2008). The spline-GARCH model for low-frequency volatility and its global macroeconomic causes. Rev. Financ. Stud..

[30] Lee C.L., Stevenson S., Lee M.L. (2018). Low-frequency volatility of real estate securities and macroeconomic risk. Account. Financ..

[31] Adrian T., Rosenberg J. (2008). Stock returns and volatility: Pricing the short-run and long-run components of market risk. J. Financ..

[32] Chernov M., Gallant A.R., Ghysels E., Tauchen G. (2003). Alternative models for stock price dynamics. J. Econom..

[33] Cotter J., Stevenson S. (2008). Modeling long memory in REITs. Real Estate Econ..

[34] Mandelbrot B.B., Van Ness J.W. (1968). Fractional Brownian Motions, Fractional Noises and Applications. SIAM Rev..

[35] Moving Average Cluster Entropy Code. https://www.dropbox.com/sh/9pfeltf2ks0ewjl/AACjuScK_gZxmyQ_mDFmGHoya?dl=0.

[36] Geometric Brownian Motion Code. https://it.mathworks.com/help/finance/gbm.html.

[37] Generalized Autoregressive Conditional Hetereskedastic Code. https://www.mathworks.com/help/econ/garch.html.

[38] Fractional Brownian Motion Code. https://project.inria.fr/fraclab/.

[39] Autoregressive Fractional Integrated Moving Average Code. https://www.mathworks.com/matlabcentral/fileexchange/25611-arfima-simulations.

